# Eukaryotic Translation Initiation Factor 4A Down-Regulation Mediates Interleukin-24-Induced Apoptosis through Inhibition of Translation

**DOI:** 10.3390/cancers10050153

**Published:** 2018-05-22

**Authors:** Xuelin Zhong, Leah Persaud, Hilal Muharam, Ashleigh Francis, Dibash Das, Bertal Huseyin Aktas, Moira Sauane

**Affiliations:** 1 Department of Biological Sciences, Herbert H. Lehman College, City University of New York, 250 Bedford Park Boulevard West, Bronx, NY 10468, USA; xzhong@gradcenter.cuny.edu (X.Z.); LEAH.PERSAUD@lehman.cuny.edu (L.P.); HILAL.MUHARAM@lc.cuny.edu (H.M.); ashleighfrancis64@gmail.com (A.F.); dd791@hunter.cuny.edu (D.D.); 2 Department of Biology, The Graduate Center, City University of New York, 365 Fifth Avenue, Room 4315, New York, NY 10016, USA; 3 Department of Medicine, Brigham and Women’s Hospital, 75 Francis Street, Boston, MA 02115, USA; huseyin_aktas@hms.harvard.edu; 4 Harvard Medical School, Laboratory for Translational Research, One Kendall Square, Building 600, 3rd Floor, Cambridge, MA 02139, USA

**Keywords:** Interleukin 24, eukaryotic initiation factor 4F complex, eukaryotic initiation factor 4A, Sigma 1 receptor, translation regulation, apoptosis

## Abstract

Dysregulated activity of helicase eIF4A drives transformation to and maintenance of cancer cell phenotype by reprogramming cellular translation. Interleukin 24 (IL-24) is a tumor-suppressing protein, which has the ability to inhibit angiogenesis, sensitize cancer cells to chemotherapy, and induce cancer cell-specific apoptosis. In this study, we found that eIF4A is inhibited by IL-24. Consequently, selective reduction of translation was observed for mRNAs harboring strong secondary structures in their 5′-untranslated regions (5′UTRs). These mRNAs encode proteins, which function in cell survival and proliferation. Consistently, overexpression of eIF4A conferred cancer cells with resistance to IL-24-induced cell death. It has been established that inhibition of eIF4A triggers mitochondrial-mediated apoptosis. We showed that IL-24 induces eIF4A-dependent mitochondrial depolarization. We also showed that IL-24 induces Sigma 1 Receptor-dependent eIF4A down-regulation and mitochondrial depolarization. Thus, the progress of apoptosis triggered by IL-24 is characterized by a complex program of changes in regulation of several initiation factors, including the eIF4A.

## 1. Introduction

Cancer cells lose the physiologic restraints on the cellular translational machinery, leading to increase in both global protein synthesis and translation of specific mRNAs that promote tumor cell survival [1]. Translation initiation plays a critical role in the physiological regulation of cell proliferation, differentiation and apoptosis. Unrestricted translation initiation not only critically contributes to the maintenance and progression of cancers but also causes malignant transformation [2,3]. Two major rate-limiting steps in translation initiation regulation are the assembly of ternary complex (TC) and eIF4F complexes [4]. Targeting translational control in general and in particular, the eIF4F translation complex, is currently at the forefront of the development a promising therapeutic strategy [5,6]. 

Dysregulated activity of helicase eIF4A drives transformation to and maintenance of cancer cell phenotype by reprogramming cellular translation. Interleukin 24 (IL-24) is a tumor-suppressing protein, which has the ability to inhibit angiogenesis, sensitize cancer cells to chemotherapy, and induce cancer cell-specific apoptosis. As proceeds via translation initiation mechanisms that are dependent either on the 5′ cap structure (m^7^GpppN, where N can be any nucleotide) or an internal ribosome entry site (IRES) [7,8]. Also, Short 5′ untranslated region (5′UTR) mRNAs are enriched with TISU (translation initiator of short 5′UTR), a 12-nucleotide element directing efficient scanning-independent translation. The majority of translation initiation events in eukaryotes are mediated through cap-dependent translation whereby the 40S ribosomal subunit is recruited to the vicinity of the mRNA 5′ cap structure by the eukaryotic initiation factor 4F (eIF4F) complex [9]. eIF4F is comprised of eIF4A (an RNA helicase), eIF4E (the cap-binding subunit), and eIF4G (a large scaffolding protein for eIF4A, eIF4E, and other initiation factors). Once assembled at the 5′ cap, the 40S ribosomal subunit in association with several initiation factors scans the 5′UTR of the mRNA until it encounters a start codon in a favorable context, followed by polypeptide synthesis. eIF4A stimulates translation of both capped and uncapped mRNAs in vitro [9]. 

Our previous studies have shown that a non-replicating adenoviral vector expressing Interleukin 24 (Ad.IL-24) induces apoptosis selectively in cancer cells, mediated through phosphorylation of eIF2α [10]. The same non-replicating adenoviral vector expression IL-24 (INGN241) was used in patients with metastatic melanoma [11]. Indeed, clinical trials in which Ad.IL-24 was administered by intratumoral injection in patients with advanced solid tumors, show promising results [12,13]. We and others have extensively investigated and reported the underlying apoptotic mechanisms of IL-24 protein treatment and Ad.IL-24 infection in preclinical studies in several cancer cells including prostate cancer cells (DU-145), melanoma (HO-1), breast cancer cells (MCF-7), and cervical cancer cells (HeLa) (reviewed in [14,15]). Our previous studies have shown that recombinant bioactive IL-24 protein as well as secreted IL-24 protein (generated from Ad.IL-24-infected cells) exerts cancer-specific killing through a mechanism identical to Ad.IL-24 infection [16]. Interleukin 24 (IL-24) is a tumor suppressor protein that is currently in phase II clinical trials [15]. We have shown that IL-24 releases calcium (Ca^2+^) from ER-stores; induces ROS and ceramide production; and induces inhibitory phosphorylation of eIF2α [10,16,17]. We also have demonstrated that Sigma 1 Receptor (Sig1R) interacts with IL-24 and that IL-24:Sig1R complex is a critical upstream signal for IL-24-induced ER stress, calcium mobilization, and notably, phosphorylation of eIF2α and apoptosis in cancer cells [18]. Consistent with our findings, Bina and colleagues have recently shown the interaction of IL-24 and Sig1R in an in silico analysis [19]. Here we show that IL-24 causes inhibition of eIF4A in a wide variety of cancer cells. To address the physiological role of eIF4A in IL-24-induced apoptosis, we used overexpression of eIF4A. We demonstrate that IL-24 induces apoptosis through eIF4A inhibition, and mitochondria dysfunction. Importantly, the translation of mRNAs bearing long and structured 5′UTRs, such as the cell cycle regulators Cdc25C, c-myc, and ornithine decarboxylase (ODC), and the survival promoting proteins Bcl-2, Mcl1, Birca5, and X-linked inhibitor of apoptosis (XIAP), was reduced as a result of down-regulation of eIF4A triggered by IL-24. These results demonstrate that eIF4A plays a major role in IL24-mediated apoptosis in cancer cells. We show that eIF4A is required for IL-24-induced apoptosis, whereby it acts by selective reduction of translation was observed for mRNAs harboring strong to moderate secondary structures in their 5′-untranslated regions (5′UTRs), including mRNAs encoding proliferation- and survival-promoting proteins. We also show that downregulation of eIF4A is required for IL-24-induced mitochondria dysfunction. Our data here provide strong evidence that Sigma 1 Receptor (Sig1R) is a critical upstream signal for IL-24-induced eIF4A down-regulation, the translation of mRNAs bearing long and structured 5′UTRs, mitochondrial dysfunction and apoptosis in cancer cells.

## 2. Results

### 2.1. IL-24-Dependent Inhibition of eIF4A Is Necessary to Mediate Apoptosis

**Figure 1:**
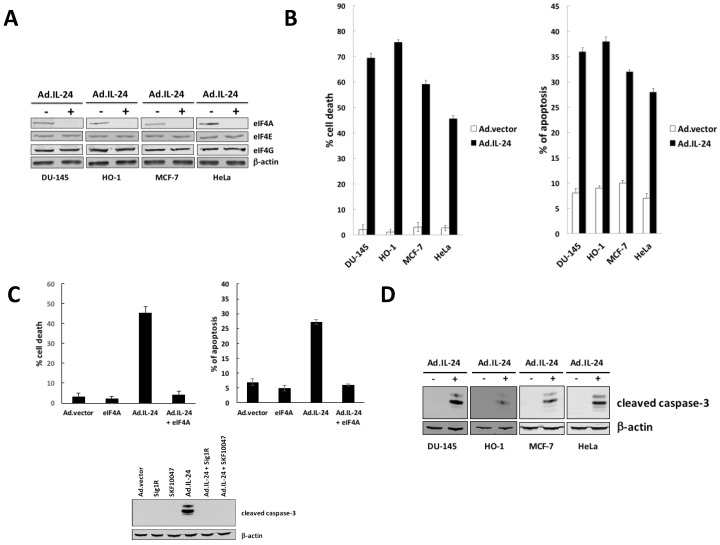
IL-24-mediatd down-regulation of eIF4A is necessary to mediate apoptosis. (**A**) Melanoma (HO-1), breast (MCF-7), prostate (DU-145) and cervical cancer cells (HeLa) were treated for 24 h with Ad.IL-24 (100 pfu per cell) or Ad.vector (100 pfu per cell). Cell extracts were subjected to Western blot analysis to detect eIF4A, eIF4G, eIF4E and β-actin protein. (**B**) Cells were treated as described in (**A**), and cell viability was determined by trypan blue exclusion assay 4 days after treatment (left panel). Numbers represent the ratio of specific treatments to values in control cells (Ad.vector). Results of cell counting by trypan blue exclusion assay are plotted as mean ± SD of three independent experiments. *, *p* < 0.001 comparted to Ad.vector. Cells were treated as described before, and then assayed for cell death using Annexin V staining a measure of apoptosis, and it was determined 48 h later by FACS analysis using the CellQuest software (Becton Dickinson). An average of three independent experiments is shown ± SD (right panel) *, *p* < 0.001 comparted to Ad.vector. (**C**) HeLa cells overexpressing eIF4A or control cells were treated with Ad.IL-24 (100 pfu per cell), and cell viability was determined by trypan blue exclusion assay. Numbers represent the ratio of specific treatments to values in control cells (Ad.vector). An average of three independent experiments is shown ± SD (left panel). *, *p* < 0.05 comparted to Ad.vector. Cells were treated as described in upper panel, and then assayed for cell death using Annexin V staining, and a measure of apoptosis was determined by FACS (right panel) *, *p* < 0.05 comparted to Ad.vector. Cells were treated as described in upper panel, cell extracts were subjected to Western blot analysis to detect cleaved caspase-3 and β-actin protein. (**D**) Melanoma (HO-1), breast (MCF-7), prostate (DU-145) and cervical cancer cells (HeLa) were treated for 24 h with Ad.IL-24 (100 pfu per cell) or Ad.vector (100 pfu per cell). Cell extracts were subjected to Western blot analysis to detect cleaved caspase-3 and β-actin protein.

Two major events are rate-limiting in translation initiation: the formation of the ternary complex (eIF2, GTP, Met-tRNAi) binds the 40S ribosomal subunit, and the assembly of the eIF4F complex on the mRNA cap [4]. We have previously demonstrated that IL-24 causes inhibition of translation, mediated through phosphorylation of eIF2α [10]. Furthermore, phosphorylated eIF2α depletes the ternary complex necessary to initiate a new round of translation [10]. As we suggested, it is plausible that the effect of IL-24 on translation regulation is also related to a broader effect of IL-24 on translation regulation, possibly involving molecular players other than eIF2α [10]. Now, we demonstrate for the first time that IL-24 inhibits eIF4A expression in prostate cancer cells (DU-145), melanoma (HO-1), breast cancer cells (MCF-7), and cervical cancer cells (HeLa) (Figure 1A). Similarly, IL-24 exposure also reduced viability of cancer cells on cell growth (Figure 1B left panel) and apoptosis (Figure 1B right panel and Figure 1D). Next, the requirement of eIF4A down-regulation for IL-24-induced apoptosis was analyzed by IL-24 treatment in cells overexpressing eIF4A. We investigated the inhibitory action of IL-24 on cell growth (Figure 1C left panel) and apoptosis (Figure 1C right panel and lower panel) on HeLa cells overexpressing eIF4A or control cells. These results show that down-regulation of eIF4A is responsible for the inhibitory effect of IL-24 on cell growth and apoptosis.

### 2.2. IL-24 Appears to Affects Translation of mRNAs with Long, but Not Short, 5′UTRs 

It has been established that eIF4A promotes the translation of mRNAs with long and structured 5′UTR features [20,21]. To confirm that IL-24 translationally down-regulates expression of long and structured 5′UTR mRNAs, HeLa cells were co-transfected with Renilla (control) and Firefly FF) luciferase reporters harboring structured 5′UTRs or unstructured 5′UTRs. The FF reporters used were: IRF7 [5′UTR]-FF construct (which is long and translated in an eIF4E-dependent manner); ATP5O [5′UTR]-FF construct, containing a 4-nt portion of the TISU element upstream of the initiation codon; ATP5O [TISU]-FF construct with a disrupted TISU element; ATP5O [5′UTR]-SL-FF construct, with a stable stem-loop structure (which is translated in an eIF4A-dependent manner); UQCC2 5′UTR construct without a TISU element; and NDUFS6 5′UTR construct with only a portion of the TISU element upstream of the initiation codon (Table 1).

**Figure 2:**
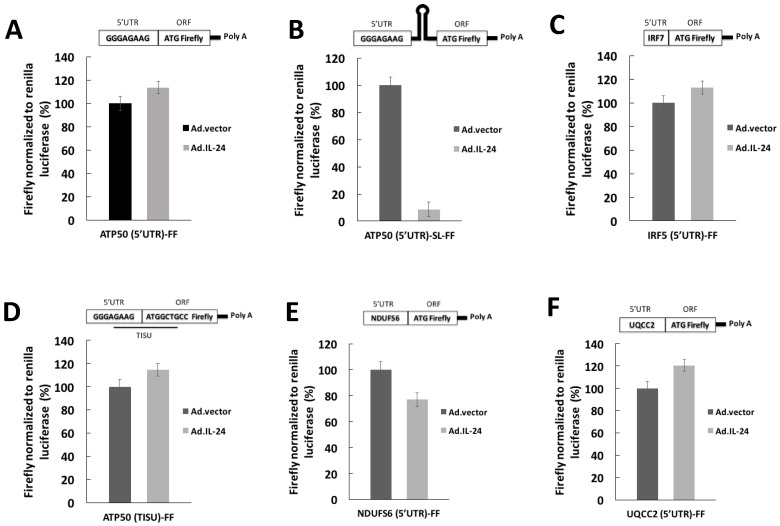
IL-24 appears to reduces translation of mRNAs harboring structured 5′UTRs. HeLa cells were transfected with firefly (FF) reporters harboring: (**A**) ATP5O 5′UTR with a proximal portion of TISU element before the initiation codon (ATP5O [5′UTR]-FF); (**B**) ATP5O 5′UTR followed by a stem-loop structure (ATP5O (5′UTR)-SL-FF); (**C**) IRF7 5′UTR (IRF7 (5;UTR)-FF); (**D**) ATP5O 5′UTR with a full TISU element (ATP5O (TISU)-FF); (**E**) NDUFS6 5′UTR (NDUFS6 (5′UTR)-FF); or (**F**) UQCC2 5′UTR (UQCC2 (5′UTR)-FF). As a control, cells were cotransfected with a Renilla reporter. Cells were treated for 24 h with Ad.IL-24 (100 pfu per cell) or Ad.vector (100 pfu per cell). Each experiment was performed in independent triplicates, each consisting of three replicates, and data are shown as mean ± SD. *p* < 0.001 comparted to Ad.vector. RLU, relative light units.

As shown in Figure 2, the main effect of treatment with IL-24 was seen on ATP5O [5′UTR]-SL-FF (Figure 2B) construct whose luciferase activity was strongly repressed, other constructs showed smaller effects. IL-24 had no effect on control luciferase. Results of these assays showed that IL-24 inhibits eIF4A expression (Figure 1A) and also IL-24 affects translation of mRNAs with long and structured 5′UTRs (Figure 2).

### 2.3. IL-24 Appears to Reduces Translation of mRNAs Harboring Structured 5′UTRs 

**Figure 3:**
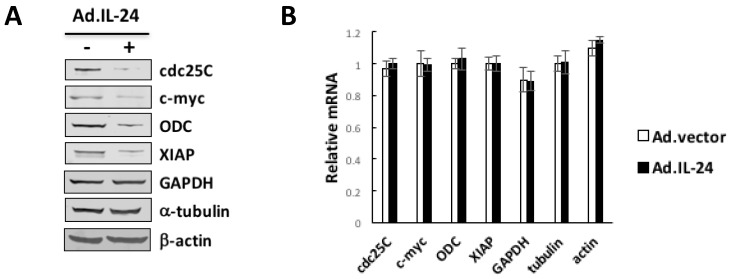
IL-24 treatment leads to reduction of mRNAs harboring structured 5′UTRs. (**A**) HeLa cells were treated with Ad.IL-24 (100 pfu per cell) or Ad.vector (100 pfu per cell) for 72 h, and lysates were prepared and probed with antibodies specific to cdc25, c-myc, ODC, XIAP, GAPDH, α-tubulin, and β-actin. (**B**) HeLa cells were incubated with Ad.IL24 (100 pfu per cell) or Ad.vector (100 pfu per cell), and expression of cdc25, c-myc, ODC, XIAP, GAPDH, α-tubulin, and β-actin mRNA was determined by real-time PCR.

Expression of most proteins bearing long and structured 5′UTRs, such as the cell cycle regulators and the survival promoting proteins is translationally controlled and is highly dependent on the activity of the helicase eIF4A that function to unwind long and structured 5′ ends of mRNAs. To determine if IL-24 translationally down-regulates such mRNAs, we performed Western blot and quantitative real-time PCR analyses of lysates from HeLa cells treated with IL-24 or control (Ad.vector). Figure 3 shows that IL-24 significantly reduced the expression of proteins that are involved in cell proliferation (e.g., Cdc25C, c-myc, and ornithine decarboxylase), and of survival promoting proteins (e.g., XIAP), whereas the expression of housekeeping proteins such as β-actin, GAPDH, and β-tubulin was not affected (Figure 3A). Although the rapid turnover of ODC, the effect of IL-24 on ODC down-regulation is not absolute. It is plausible that this effect involves other molecular players such as antizyme-1, and localization of ODC in non-synchronized HeLa cells. In HeLa cells that overexpress eIF4A, IL-24 did not affect the levels of Cdc25C, c-myc, ornithine decarboxylase, and XIAP expression (Figure 5D). Down-regulation of most proteins was likely translational because IL-24 has minimal effects on the levels of the respective mRNAs (Figure 3B). These findings are consistent with the view that inhibitors of eIF4A preferentially affect the expression of proteins involved in cell proliferation and survival. 

### 2.4. IL-24 Treatment Leads to Mitochondrial Dysfunction 

**Figure 4:**
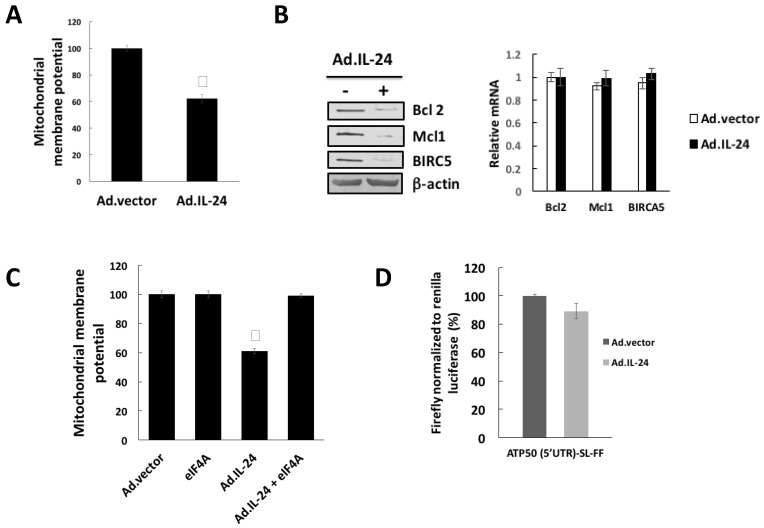
IL-24 treatment leads to mitochondrial dysfunction. (**A**) HeLa cells were treated with Ad.IL-24 (100 pfu per cell) or Ad.vector (100 pfu per cell) for 72 h. Mitochondrial membrane potential (MMP) was analyzed by monitoring TMRE fluorescence intensity. Results are presented as mean ± SD (*n* = 3) *, *p* < 0.05 comparted to Ad.vector. (**B**) HeLa cells were treated with Ad.IL-24 (100 pfu per cell) or Ad.vector (100 pfu per cell) for 72 h, and lysates were prepared and probed with antibodies specific to Bcl2, Mcl1, BIRC5, and β-actin (Left panel). HeLa cells were incubated with Ad.IL24 (100 pfu per cell) or Ad.vector (100 pfu per cell), and expression of Bcl2, Mcl1, BIRC5, and β-actin mRNA was determined by real-time PCR (right panel). (**C**) HeLa cells overexpressed eIF4A or control cells were treated with Ad.IL-24 (100 pfu per cell) or Ad.vector (100 pfu per cell) for 72 h. Mitochondrial membrane potential (MMP) was analyzed by monitoring TMRE fluorescence intensity. Results are presented as mean ± SD (*n* = 3) *, *p* < 0.05 compared to Ad.vector. (**D**) HeLa cells overexpressing eIF4A cells were transfected with firefly (FF) reporters harboring ATP5O 5′UTR followed by a stem-loop structure (ATP5O (5′UTR)-SL-FF). As a control, cells were cotransfected with a Renilla reporter. Cells were treated for 24 h with Ad.IL-24 (100 pfu per cell) or Ad.vector (100 pfu per cell). Luminescence was monitored 48 h post-transfection. Data for Firefly luminescence normalized to Renilla luminescence. Each experiment was performed in independent triplicates, each consisting of three replicates, and data are shown as mean ± SD. *, *p* < 0.005 comparted to Ad.vector. RLU, relative light units.

It has been established that inhibition of eIF4A triggers mitochondrial-mediated apoptosis by inhibiting translation of mRNAs with long 5′UTRs encoding proteins that maintain mitochondrial structural integrity (e.g., Bcl-2, Mcl1, and BIRC5) [22,23]. Therefore, we investigated the effects of IL-24 on mitochondrial function. HeLa cells were treated with IL-24 for 72 h and mitochondrial membrane potential was measured using tetramethylrhodamine ethyl ester (TMRE), which can only accumulate in mitochondria when they are active. We found that IL-24 treatment induced depolarization of mitochondria (Figure 4A) as well as down-regulation of proteins implicated in the maintenance of the integrity of the mitochondrial outer membrane (Figure 4B left panel). Down-regulation of proteins implicated in the maintenance of the integrity of the mitochondrial outer membrane was likely translational because IL-24 has minimal effects on the levels of the respective mRNAs (Figure 4B right panel). The requirement of eIF4A down-regulation for IL-24-induced mitochondria dysfunction was analyzed by IL-24 treatment together with overexpression of eIF4A. The action of IL-24 on mitochondria dysfunction was tested in HeLa cells overexpressing eIF4A (Figure 4C). These results show that inhibition of eIF4A expression is responsible for the effect of IL-24 on mitochondria dysfunction. 

### 2.5. IL-24-Dependent Sigma 1 Receptor (Sig1R) Mediate eIF4A Down-Regulation, Translation of mRNAs Harboring Structured 5′UTRs and Mitochondrial Dysfunction 

**Figure 5:**
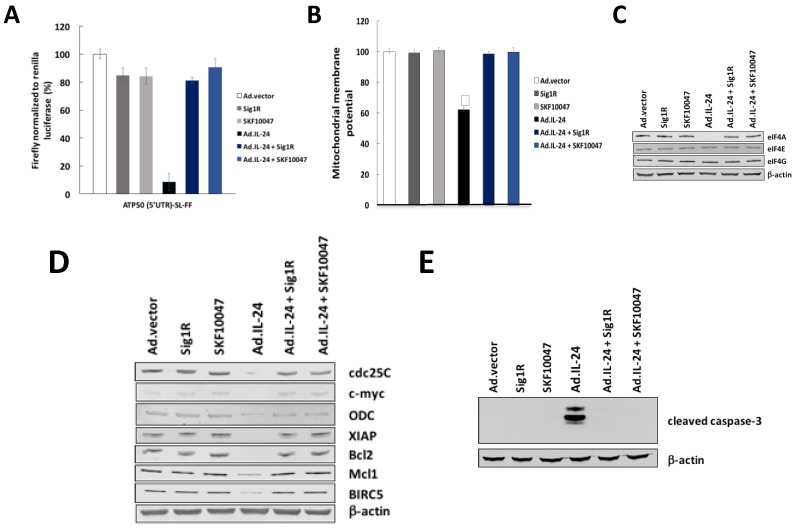
IL-24-dependent Sigma 1 Receptor (Sig1R) mediate eIF4A down-regulation, translation of mRNAs harboring structured 5′UTRs and mitochondrial dysfunction. (**A**) HeLa cells were transfected with firefly (FF) reporter harboring ATP5O 5′UTR followed by a stem-loop structure (ATP5O [5′UTR]-SL-FF). Cells were cotransfected with a Renilla reporter. Cells were incubated with the Sig1R agonist (SKF10047) or overexpression of Sig1R with or without Ad.IL-24 (100 pfu per cell). Data for firefly luminescence normalized to Renilla luminescence. Each experiment was performed in independent triplicates, each consisting of three replicates, and data are shown as mean ± SD. *, *p* < 0.05. RLU, relative light units. (**B**) HeLa cells were treated as described in (**A**). Mitochondrial membrane potential (MMP) was analyzed by monitoring TMRE fluorescence intensity. Results are presented as mean ± SD (*n* = 3). *, *p* < 0.05. (**C**) HeLa cells were incubated with the indicated conditions. Cells were collected, protein purified, and subjected to western blot analysis to detect eIF4A, eIF4G and eIF4E protein. (**D**) HeLa cells were treated as described in (**A**), and lysates were prepared and probed with antibodies specific to cdc25, c-myc, ODC, XIAP, Bcl2, Mcl1, BIRC5, and β-actin. (**E**) HeLa cells were treated as described in A, and lysates were prepared and probed with antibodies specific to cleaved caspase-3 and β-actin.

Sigma 1 Receptor (Sig1R) is a protein chaperone, through whose regulation by specific ligands can have either cytoprotective or cytotoxic effects [24]. Sig1R agonists bind to Sig1R and promote cellular survival by preventing oxidative stress, while conversely, Sig1R antagonists, bind to Sig1R and inhibit tumor cell survival and induce apoptosis [15]. We have demonstrated that Sig1R is the common upstream initial signal transduction molecule involved in IL-24-induced cancer-specific apoptosis [18]. We have determined that IL-24 action in cancer cells is mediated by an antagonistic effect of IL-24 on Sig1R. Sig1R antagonist-mediated cell death is inhibited by the prototypic Sig1R agonist SKF10047 or by overexpression of Sig1R [15,24]. Therefore, we determined the possible role of Sig1R in the effect of IL-24 on eIF4A down-regulation as well as mitochondria dysfunction. HeLa cells were transfected with Sig1R-expressing construct or treated with Sig1R agonist (SKF10047), and the action of IL-24 on eIF4A down-regulation as well as mitochondria dysfunction was tested. As shown in Figure 5, Sig1R agonist SKF10047 blocks Ad.IL-24-mediated eIF4A down-regulation (Figure 5A,C), blocks mitochondrial dysfunction (Figure 5B) as well as translation of mRNAs with long 5′UTRs (Figure 5D). The requirement of Sig1R for IL-24-induced apoptosis was analyzed by IL-24 treatment together with Sig1R agonist SKF10047 or by overexpression of Sig1R. These results show that IL-24-mediated eIF4A down-regulation under the control of Sigma 1 Receptor as important mediators of apoptosis, translation of mRNAs with long 5′UTRs as well as mitochondrial dysfunction.

## 3. Discussion

Aberrant translation has a profound impact on malignancy [1,25,26,27,28,29]. The work reported here provides a previously unrecognized mode of inhibition of translation initiation whereby IL-24 inhibited eIF4A to induce apoptosis in cancer cells. Specifically, we demonstrated that IL-24 treatment preferentially inhibits the translation of mRNAs possessing long and structured 5′UTRs at the initiation step. In agreement with this model, our data demonstrate that IL-24 treatment inhibits eIF4A protein expression, and therefore inhibits the translation of mRNAs harboring structured 5′UTRs but not mRNAs with relatively unstructured 5′UTRs. We show that through this mechanism, IL-24 targets the expression of specific mRNAs harboring strong secondary structures in their 5′UTRs. These mRNAs encode proteins, which function in survival (XIAP and Bcl-2) and in cell proliferation (Cdc25C, ODC, and c-myc). Importantly, we show that overexpression of eIF4A conferred cancer cells with resistance to IL-24-induced cell death and targets the expression of highly structured 5′UTRs mRNAs. Thus, we provide the first direct evidence for translational control of gene-specific expression by IL-24 through the regulation of eIF4A. This is consistent with the paradigm in the field of translational regulation of gene expression, that down-regulation of eIF4A seems to be a promising cancer therapeutic approach with a pronounced effect on the translation of mRNAs encoding proteins which are important for proliferation, metabolism and survival, providing cancer cells a selective advantage [21,30,31,32,33].

How the two major regulatory branches that regulate translation, ternary complex and eIF4F assembly, are coordinated remains largely unknown. Interestingly, Gandin et al. demonstrated a coordinated regulation of eIF2α and eIF4E via CK2 and mTORC1 [22]. Our group recently demonstrated the role of IL-24 in inhibition of translation, mediated through phosphorylation of eIF2α [10]. Consistent with this and with the new results reported here, both the ternary complex formation and eIF4F complex branches are inhibited by IL-24. It is not clear yet if both of these facets of translation initiation can be modulated, together or separately by one or more upstream signals triggered by IL-24. To characterize the signal transduction pathway responsible for the down-regulation eIF4A by IL-24, we focused on the upstream pathways involved in IL-24-induced apoptosis. Our data here provide strong evidence that the IL-24 down-regulation of eIF4A expression is Sig1R-dependent (Figure 5). Also, in this study, we demonstrate that IL-24 induces eIF4A-dependent and Sig1R-dependent mitochondrial depolarization (Figure 5). Therefore, it is plausible that Sig1R coordinates TC and eIF4F complex assembly triggered by IL-24. Future research is required to delineate the mechanisms by which Sig1R down-regulates eIF4A expression triggered by IL-24 and to investigate the mechanisms by which Sig1R mediated through both phosphorylation of eIF2α and de-phosphorylation of 4E-BP1. 

## 4. Materials and Methods

### 4.1. Cell Lines and Reagents

All cell lines were purchased from American Type Culture Collection (ATCC, Manassas, VA, USA), maintained per ATCC protocols and utilized within six months of thawing each vial. Human cervical carcinoma HeLa cells expressing either empty vector (HeLa control) or eIF4A (HeLa eIF4A) stable transfected cells were described previously [34,35]. Briefly, cells were seeded at a density of 2 × 10^5^ in 60 mm and transfected using the Qiagen transfectamine transfection kit (Qiagen, Stanford Valencia, CA, USA). Cell viability was analyzed by trypan blue exclusion assay. Cells were trypsinized and an aliquot suspended 1:1 volume with 0.4% trypan blue. Total cell numbers and cell viability counts were assessed using a hemocytometer by light microscopy. 

### 4.2. Western Blot Analysis

Protein extracts were prepared with RIPA buffer containing a mixture of protease inhibitors as described. Briefly, fifty micrograms of protein were applied to a 12% SDS/PAGE and transferred to nitrocellulose membranes. Membranes were incubated with Odyssey blocking buffer (LI-COR Odyssey Biosciences, Lincoln, NE, USA) prior to incubation with polyclonal or monoclonal antibodies to eIF4A, eIF4E, eIF4G, Cdc25C, c-myc, ornithine decarboxylase, Bcl-2, X-linked inhibitor of apoptosis, Mcl1, BIRCA5, α-tubulin, Glyceraldehyde 3-phosphate dehydrogenase (GAPDH), cleaved caspase-3, and β-actin overnight at 4 °C. Goat anti-rabbit IgG (H+L) 800 CW, goat anti-rabbit (680 RD) and/or goat anti-mouse (H+L) was applied for 60 min at room temperature (1:25,000, LI-COR Odyssey Biosciences) prior to washing with 1X Phosphate Buffered Saline Tween-20 (PBS-T). Visualization and quantification was carried out with the LI-COR Odyssey scanner and software (LI-COR Biosciences Lincoln, NE, USA).

### 4.3. Generation of Luciferase Reporters and Luciferase Assays

Luciferase reporters (kindly provided by Dr. Ivan Topisirovic) and luciferase assays were described previously [22]. HeLa cells were seeded in a 10 cm Petri dish and next day transfected using 30 µL of Lipofectamine 2000 (Invitrogen, Carlsbad, CA, USA), 600 ng of control Renilla (kindly provided by Drs. Yuri Svitkin and Nahum Sonenberg) and 1.2 µg of the firefly luciferase constructs. The next day, cells were seeded in triplicate in a 12-well plate and translation of the indicated reporters was monitored 48 h post-transfection using Dual Luciferase Assay System (Promega, Madison, WI, USA) according to the manufacturer’s instructions.

### 4.4. Annexin V Binding Assay

Cells were trypsinized, washed once with complete medium and PBS, resuspended in 0.5 mL of binding buffer containing 2.5 mmol/L CaCl_2_, and stained with allophycocyanin-labeled Annexin V (Becton Dickinson Biosciences, Palo Alto, CA, USA) and propidium iodide (PI) for 15 min at room temperature. Flow cytometry assays were performed as previously described [10].

### 4.5. RT-PCR

Total RNA was isolated by using the RNAeasy kit (Qiagen). Reverse transcription (RT) was performed on 5 ng of total RNA with an oligo(dT) primer. cDNA corresponding to 5 ng of total RNA was amplified for 35 cycles by PCR with specific primers as previously described [17]. Sequence-validated QuantiTec probes for eIF4A, eIF4B, eIF4G, Cdc25C, c-myc, ornithine decarboxylase, Bcl-2, Mcl1, Birca5, X-linked inhibitor of apoptosis, Glyceraldehyde 3-phosphate dehydrogenase (GAPDH) and β-actin purchased from Qiagen Bio-technology were used for these mRNAs.

### 4.6. Mitochondrial Membrane Potential

Mitochondrial membrane potential was monitored in intact cells by confocal microscopy using tetramethylrhodamine ethyl ester (TMRE), which can only accumulate in mitochondria when they are active. HeLa cells were seeded on 25 mm cover slips at a density of 600,000 cells/well, grown for 48 h, and then incubated with JC1 (10 μg/mL) for 2 h. The cover slip was washed twice and mounted in a cell chamber (ALA Scientific Instruments, Westbury, NY, USA). 

### 4.7. Statistical Analysis

Experiments were performed at least in duplicate or triplicate, and data represent the average of three independent experiments. Statistical analysis was performed using Student *t* test. *p* values less than or equal to 0.05 were considered significant. Experiments shown are the means of multiple individual points from multiple experiments (±SEM).

## 5. Conclusions

These results demonstrate a previously unrecognized role of IL-24 in inhibition of translation, mediated through eIF4A down-regulation, and identifies IL-24-mediated eIF4A down-regulation under the control of Sigma 1 Receptor as important mediators of apoptosis, translation of mRNAs with long 5′UTRs as well as mitochondrial dysfunction.

## Figures and Tables

**Table 1:** Primers used to construct luciferase reporter plasmids. List of primers used to generate luciferase reporter constructs.

Name	Sequence
ATP5O 5’UTR-FF (forward)	5’-TACCGAGCTCGGATCCAAGGGAGAAGATGGAAGACGCCAA-3’
ATP5O 5’UTR-FF (reverse)	5’-TTGGCGTCTTCCATCTTCTCCCTTGGATCCGAGCTCGGTA-3’
UQCC2 5’UTR-FF (forward)	5’-TACCGAGCTCGGATCCAAGGGGCCCAAGATGGAAGACGCCAA-3’
UQCC2 5’UTR-FF (reverse)	5’-TTGGCGTCTTCCATCTTGGGCCCCTTGGATCCGAGCTCGGTA-3’
NDUFS6 5’UTR-FF (forward)	5’-TACCGAGCTCGGATCCAAGGGTCAAAGGCCAGCGGCGCAAAATGGAAGACGCCAA-3’
NDUFS6 5’UTR-FF (reverse)	5’-TTGGCGTCTTCCATTTTGCGCCGCTGGCCTTTGACCCTTGGATCCGAGCTCGGTA-3’
ATP5O (TISU)-FF (forward)	5’-CGGATCCAAGGGAGAAGATGGCTGCCGCCAAAAACATAAAGAAAGG-3’
ATP5O (TISU)-FF (reverse)	5’-CCTTTCTTTATGTTTTTGGCGGCAGCCATCTTCTCCCTTGGATCCG-3’
ATP5O (5’UTR)-SL-FF (forward)	5’-AGCTAAGCTTGGGAGAAGGTCCACCACGGCCGATATCACGGCCGTGGTGGACGGATCCGACT-3’
ATP5O (5’UTR)-SL-FF (reverse)	5’-GACTGGATCCGTCCACCACGGCCGTGATATCGGCCGTGGTGGACCTTCTCCCAAGCTTAGCT-3’
